# Stigma and Media Charter: A Belgian Initiative to Combat Psychiatric Stigma in Public Media

**DOI:** 10.1192/j.eurpsy.2026.10750

**Published:** 2026-07-31

**Authors:** E. Thys, K. C. Catthoor

**Affiliations:** 1 Psychiatry, UPC KU Leuven, Leuven; 2 Psychiatry, PSC Elsene, Brussels; 3 Psychiatry, ZAS PZ Stuivenberg, Antwerp, Belgium

## Abstract

**Introduction:**

Psychiatric stigma—defined as negative attitudes, stereotypes, and discrimination directed toward individuals with mental health conditions—remains a significant burden that obstructs both personal and social recovery. A key contributor to this stigma is the manner in which public media portray psychiatric conditions and those affected by them. Our research indicates that problematic representations persist, although there are also signs of a gradually more informed and understanding approach within the media.

**Objectives:**

To reduce stigma by introducing the Charter on Stigma and the Media and seeking the widest possible commitment from media organizations.

**Methods:**

In collaboration with the Flemish Association of Psychiatrists (FAP), the Estates General of Mental Health (EGMH), and the Flemish Association of Journalists (VVJ), we developed a charter comprising ten recommendations for non-stigmatizing reporting in public media (television, radio, newspapers, and their online platforms). These recommendations are based on the Flemish Code of Journalistic Ethics. The charter also includes general information on the most common psychiatric conditions and provides reliable sources for accurate information.

**Results:**

As of September 2025 (to be updated), a majority of print media outlets, along with several radio and television stations, have signed the charter. Each formal signing received media coverage; notably, the first was held in the presence of two ministers and a representative of a third. A number of French-language Belgian media outlets have meanwhile also pledged their commitment to the charter.

Updated figures will be included in the final presentation.

**Conclusions:**

This development represents a promising step toward reducing stigmatization. Looking ahead, two key tasks remain:

1. Continuously monitoring the signatory media’s adherence to the charter.

2. Expanding efforts to engage all media outlets to sign the charter.

**Disclosure of Interest:**

None Declared

